# The secular trend of enterovirus A71 after the implementation of preventive measures in Taiwan

**DOI:** 10.1186/s12889-022-13916-0

**Published:** 2022-08-04

**Authors:** Ya-Li Hu, Chiu-Mei Chen, En-Tzu Wang, Hung-Wei Kuo, Wei-Liang Shih, Chi-Tai Fang, Ding-Ping Liu, Luan-Yin Chang

**Affiliations:** 1 Department of Pediatrics, Cathay General Hospital, Taipei, Taiwan; 2 Department of Pediatrics, National Taiwan University Hospital and National Taiwan University College of Medicine, No. 8, Chung Shan S. Rd., Taipei, 10041 Taiwan; 3 Institute of Epidemiology and Preventive Medicine, College of Public Health, National Taiwan University and Infectious Diseases Research and Education Center, Ministry of Health and Welfare and National Taiwan University, Taipei, Taiwan; 4 Epidemic Intelligence Center, Centers for Disease Control, No. 6, Linsen S. Rd., Taipei, 10050 Taiwan

**Keywords:** Enterovirus A71, Complication, Epidemiology, Surveillance system, Infection control

## Abstract

**Background:**

Enterovirus A71 (EV A71) is one of the most important enteroviruses related to morbidity and mortality in children worldwide. This study aimed to analyse the secular trend of EV A71 in Taiwan from 1998 to 2020 and to evaluate the effectiveness of infection control measures.

**Methods:**

We collected the epidemiological data of EV A71 from disease surveillance systems in Taiwan. We analysed the association between the secular trend of EV A71 and preventive measures such as hand washing, case isolation, and suspension of classes.

**Results:**

The incidence of enterovirus infections with severe complications (EVSC) decreased from 16.25 per 100,000 children under six in 1998 to less than 9.73 per 100,000 children under six after 2012 (*P* = 0.0022). The mortality rate also decreased significantly, from 3.52 per 100,000 children under six in 1998 to 0 per 100,000 children under six in 2020 (*P* < 0.0001). The numbers of EVSC and fatalities were significantly higher in the years when EV A71 accounted for more than 10% of the annual predominant serotypes (*p* < 0.05). After the implementation of many non-pharmaceutical interventions in 2012, the incidence of EVSC and mortality rate decreased significantly (*p* < 0.001).

**Conclusions:**

After implementing active enterovirus surveillance and preventive measures, we found that the incidence of EVSC and fatalities due to EV A71 in Taiwan decreased significantly from 1998 to 2020. Continuous surveillance and strengthened infection control policies are still needed in the future.

## Background

Enterovirus A71 (EV A71) was first isolated in California (USA) in 1969 [1]. Epidemic outbreaks occurred in succession in other countries [2–8]. Most EV A71 infections presented as hand, foot, and mouth disease (HFMD), which accounted for 80 to 90% of the cases [9]. Severe complications, including brainstem encephalitis, cardiopulmonary failure, and acute flaccid paralysis, may occur within two to 7 days after symptom onset [9, 10]. Moreover, several hundreds to thousands of people with a severe case of the disease die every year in some countries [11, 12]. Apart from a high mortality rate, EV A71 could cause long-term neurological sequelae and cognitive impairment [10].

Before 1997, two large outbreaks associated with a high case fatality rate occurred in Bulgaria in 1975, with 44 deaths [3], and Hungary in 1978, with 45 deaths [4]. After that, EV A71 circulated and caused outbreaks in a cyclical pattern every two to 3 years, mainly in the Asia-Pacific region [13, 14]. Several epidemic situations leading to child death have been reported in many Asian countries such as Malaysia [5], Taiwan [6], Vietnam [11], China [12], and Cambodia [14, 15]. On the other hand, one retrospective surveillance study conducted in 24 European and European Economic Area countries also found that EV A71 accounted for 6% of the isolated enteroviruses from 19 countries, mostly Spain and Germany, during 2015–2017 [16]. In contrast to the outbreaks of HFMD in Asia, most patients with EV A71 in this study presented with neurological symptoms. Therefore, it is of the utmost importance to control EV A71 outbreaks for child welfare.

The first large outbreak of EV A71 in Taiwan occurred in 1998 and caused 78 fatal paediatric cases [6]. According to the seroepidemiological study in Taiwan, EV A71 infection did occur before 1997. Nevertheless, some severe cases might not be recognized at that time [9, 17]. Low seroconversion rate of EV A71 during 1994 and 1997 indicated that there were many susceptible hosts and led to the outbreak in 1998 [9, 17]. EV A71 continues to circulate in Taiwan and cause outbreaks every three to 5 years. There were more than 20 children died due to EV A71 infection in both 2000 and 2001 in Taiwan [18]. Another nationwide epidemic also occurred in 2012 but low morality was observed [19]. Taiwan established multiple diseases surveillance systems for enteroviruses and a medical network for managing children with enterovirus infections with severe complications (EVSC) after the first large EV A71 outbreak in 1998. Several preventive measures, including suspension of classes, patient isolation, and public health education, have been promoted in Taiwan. In this study, we aimed to investigate the secular trend of EV A71 in Taiwan from 1998 to 2020 and to evaluate the effectiveness of the above preventive measures.

## Methods

### Background of Taiwan surveillance systems

The Taiwan Centers for Disease Control (CDC) established four surveillance systems: a national notifiable disease surveillance system, a real-time outbreak and disease surveillance system (RODS), a viral laboratory surveillance system and a school-based surveillance system. EVSC has been a formal notifiable disease in Taiwan since 1999. For EVSC, pediatric patients with any of the following three criteria should be reported to the CDC within 1 week: (1). HFMD or herpangina accompanied with myoclonic jerk or complicated with encephalitis, acute flaccid paralysis, myocarditis, cardiopulmonary failure or sepsis syndrome, (2). a respiratory tract infection with brain stem encephalitis or acute flaccid myelitis, and (3). infants younger than 3 months of age with myocarditis, hepatitis, encephalitis, thrombocytopenia and multi-organ failure. By doing this, the national notifiable disease surveillance system could monitor the epidemic trend of severe enterovirus infection.

RODS was established in 2007 and includes a total of 175 emergency departments with a current coverage rate of 85–90%. The National Health Insurance collaborated with the Taiwan CDC to monitor common enterovirus infections without complications in 2009, so both the National Health Insurance and RODS record the number of EV patients who visited the emergency or outpatient department.

There is a viral laboratory surveillance system that includes a contract laboratory for viral isolation and a laboratory automated reporting system. The Taiwan CDC integrated viral contract laboratories in March 1999 for enterovirus isolation, contributing to the understanding of serotype distribution and epidemic changes in enterovirus in the community. The laboratory automated reporting system was established to collect more real-time data in 2013. A total of 66 hospitals including 21 medical centres joined the laboratory automated reporting system and uploaded data on enterovirus isolation, serology and polymerase chain reaction test results automatically once positives were detected.

A school-based surveillance system was established to monitor enterovirus infections among kindergarteners and elementary school students in 2002, and the geographical coverage for this system is 99% at present. In addition, a cluster reporting system enrolled baby rooms, neonatal observation rooms, infant daycare centres, and postpartum care centres in order to report enterovirus cases among neonates and infants.

### Study design and ethical approval

We collected the epidemiological data of enterovirus infection from 1998 to 2020 from the four main Taiwanese surveillance systems. We analysed the association between the secular trend of EV A71 and the preventive measures and policies in different years, which are described in the following text. This study was approved by the National Taiwan University Hospital Institutional Review Board (202106180RIND).

### Observed prevention measures and medical network

Three important prevention measures including hand washing, case isolation and suspension of classes were implemented. Taiwan has promoted hand washing since 1998, when the first outbreaks of EV A71 occurred. Public health education and propaganda also focused on the importance of cleaning and disinfecting the environment to decrease the incidence of household transmission, especially during the epidemic.

To prevent transmission and clusters in preschool educational institutions, children must stay at home for one to 2 weeks if they have HFMD or herpangina. Taiwan also implemented isolation measures such as droplet and contact isolation in medical institutions to prevent nosocomial infections if the patients were hospitalized.

To reduce the risk of EVSC clusters, suspension of classes has been recommended for high-risk groups, such as children in preschool and daycare institutions. The class suspension criteria were first formulated in 2004 and modified in 2014 as the following: (1). two cases of enterovirus infection in the same class within 1 week when there was an epidemic of EV A71, and (2). two cases of enterovirus infection in the same class within 1 week when there were EV A71 cases or EVSC cases in the local district without EV A71 epidemic in Taiwan. With either one of the above 2 criteria, classes have to be suspended for 1 week.

After the large outbreaks of EV A71 in 1998, Taiwan established the system of EVSC-responsible hospitals. Severe cases would be transferred to medical centres for further management. In 2008, the Taiwan CDC renamed the 6 regional commanders, 8 to 12 viral contract laboratories and 76 responsible hospitals as a medical network for EVSC. The medical network was created to improve the quality of medical care for patients infected with enterovirus. Consultation channels staffed by clinical professionals were established, and they provided clinical health care consultations and guidelines for treating enterovirus complications. Responsible hospitals oversaw patients diagnosed with EVSC.

Taiwan established practical treatment guidelines for the stage-based management of EV A71 infections as well as neonatal enterovirus infections and acute flaccid myelitis in 2000 [20, 21]. These materials are provided on the Taiwan CDC website and updated periodically. There were also annual medical education programmes and workshops to enhance medical personnel’s skills in treating the disease, improving treatment quality, and reducing mortality rates and the incidence of sequelae.

### Data analysis and effectiveness measurement

We used a chart to summarize the case numbers of EVSC, fatalities, and the predominant serotypes over the past 22 years. Figures describing the number of emergency and outpatient visits for enterovirus infection, and enterovirus serotypes distribution were generated by using the data from RODS, National Health Insurance and laboratory surveillance system. Continuous non-normal distributed variables were analysed using the Mann-Whitney U test. The incidence of EVSC and mortality rate were calculated per 100,000 children under six. Negative binomial regression models were used to assess the secular trends and average annual percent change (AAPC) of the incidence of EVSC and mortality rate by using the observed numbers of EVSC and fatal cases as the outcome and the natural logarithm of the corresponding denominator (person-years at risk) as the offset. The unit of observation was the incidence rate and mortality rate in a single calendar year. Negative binomial regression was used because most models in this study showed overdispersion features, which were checked using the deviance divided by its degrees of freedom under a Poisson regression model. We used chi-square test to compare the incidences of EVSC and mortality rates before 2012 and after 2013 to evaluate the effectiveness of preventive measures which had been implemented in or before 2012. Statistical analysis was performed with SPSS v22.0 software (IBM United States Software). A *P*-value < 0.05 was considered statistically significant.

## Results

### The incidence of severe EV infection and fatality among children under six years of age from 1998 to 2020

**Fig. 1:**
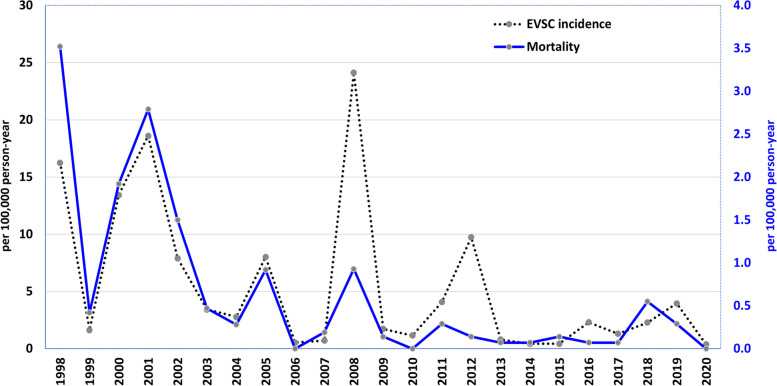
The incidence of enterovirus infection with severe complications and mortality in children under six-year-old, 1998–2020. The incidence of enterovirus infection with severe complications (EVSC) and mortality rate decreased significantly from 1998 to 2020. The average annual percentage change (AAPC) for the incidence of EVSC was −9.19% (95%CI −3.40, −14.64, *P* = 0.0022). The AAPC for mortality in children younger than six years of age was −12.81% (95%CI −7.73, −17.63, *P* < 0.0001)

The incidence of EVSC and mortality rate among children under 6 years of age are shown in Fig. 1. Negative binomial regression revealed that the incidence of EVSC declined significantly from 1998 to 2020 (AAPC = − 9.19%, *P* = 0.002). The EVSC incidence was 16.25 per 100,000 children under 6 years of age in 1998; this number reached a peak of 24.01 per 100,000 children under 6 years of age after 2009. The mortality rate also decreased significantly, from 3.52 per 100,000 children under 6 years of age in 1998 to 0 per 100,000 children under 6 years of age in 2020 (AAPC = − 12.8%, *P* < 0.0001).

### Emergency and outpatient visits for patients with hand-foot-mouth disease and herpangina

**Fig. 2:**
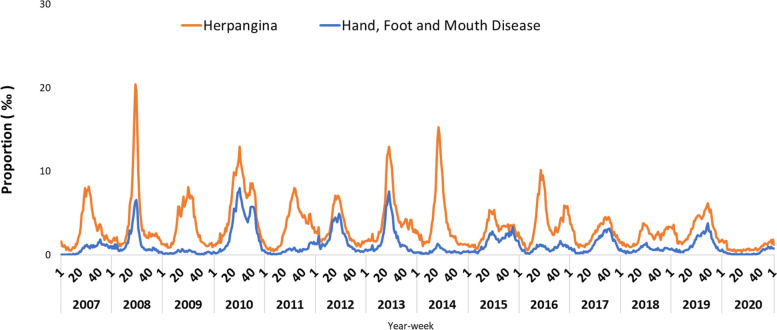
Proportion of weekly emergency visits for hand, foot and mouth disease and herpangina, 2007–2020. The peak proportion of weekly emergency visits for HFMD and herpangina occurred between week 20 and week 40 each year. The number of individuals with herpangina was higher than the number of individuals with HFMD every year. The peak annual proportion of emergency visits for enterovirus infection was less than 10% after 2017

**Fig. 3:**
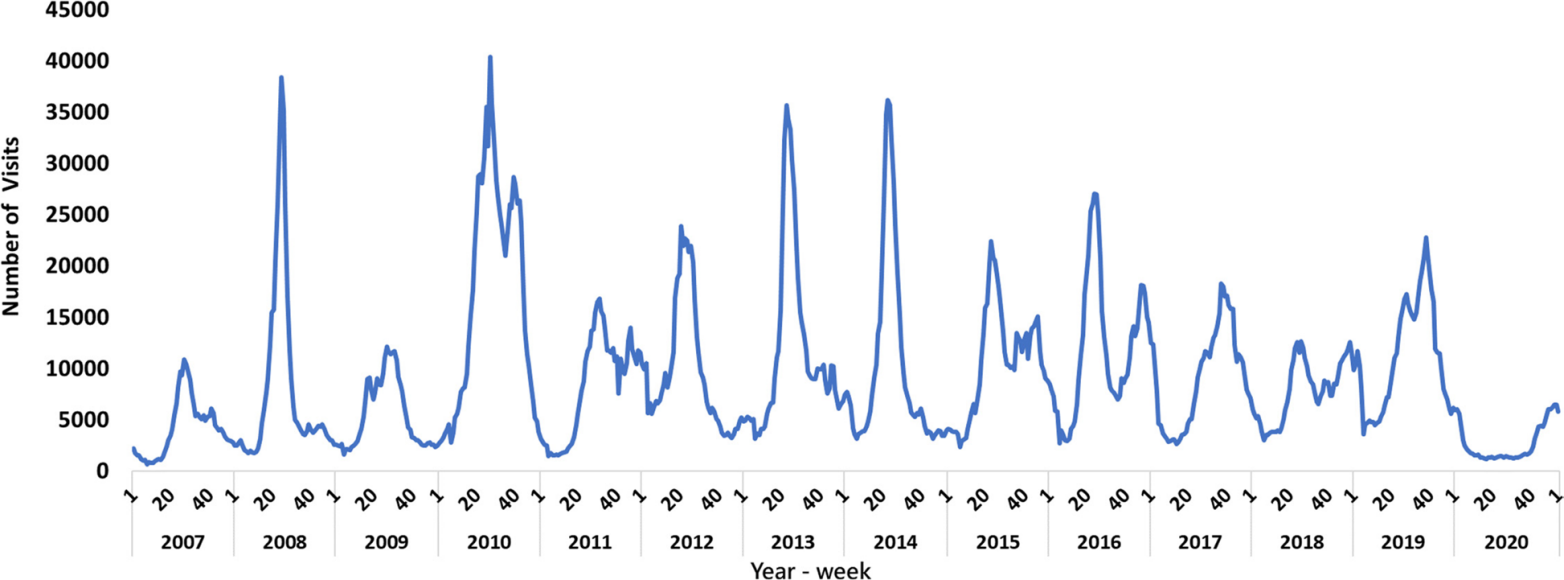
Number of weekly outpatient and emergency visits for enterovirus infection in Taiwan, 2007–2020. The number of outpatient and emergency visits for enterovirus infection showed a seasonal distribution. The peak occurred between week 20 and week 40 annually. The number of outpatient and emergency visits for enterovirus infection reached the lowest point in 2020

The proportion of emergency visits for HFMD and herpangina from RODS during 2007 and 2020 is shown in Fig. 2. According to National Health Insurance database, the number of both emergency and outpatient visits for HFMD and herpangina is shown in Fig. 3. The peak proportion of emergency visits occurred between week 20 and week 40 each year. The proportion of herpangina was the highest, up to 20%, in 2008. Both the proportion of emergency visits for HFMD and herpangina decreased to less than 10% between 2017 and 2020. As shown in Fig. 2, there were epidemic surges of enterovirus infection, with more than 35,000 outpatient and emergency visits per week in 2008, 2010, 2013 and 2014. In contrast, the numbers of weekly outpatient and emergency visits for enterovirus infection were below the usual level in 2020, and the mean numbers of outpatient and emergency visits for enterovirus infection were less than 2500 per week as Fig. 3 shows.

### The trend of EVSC and fatal cases after the implementation of surveillance and preventive measures

**Fig. 4:**
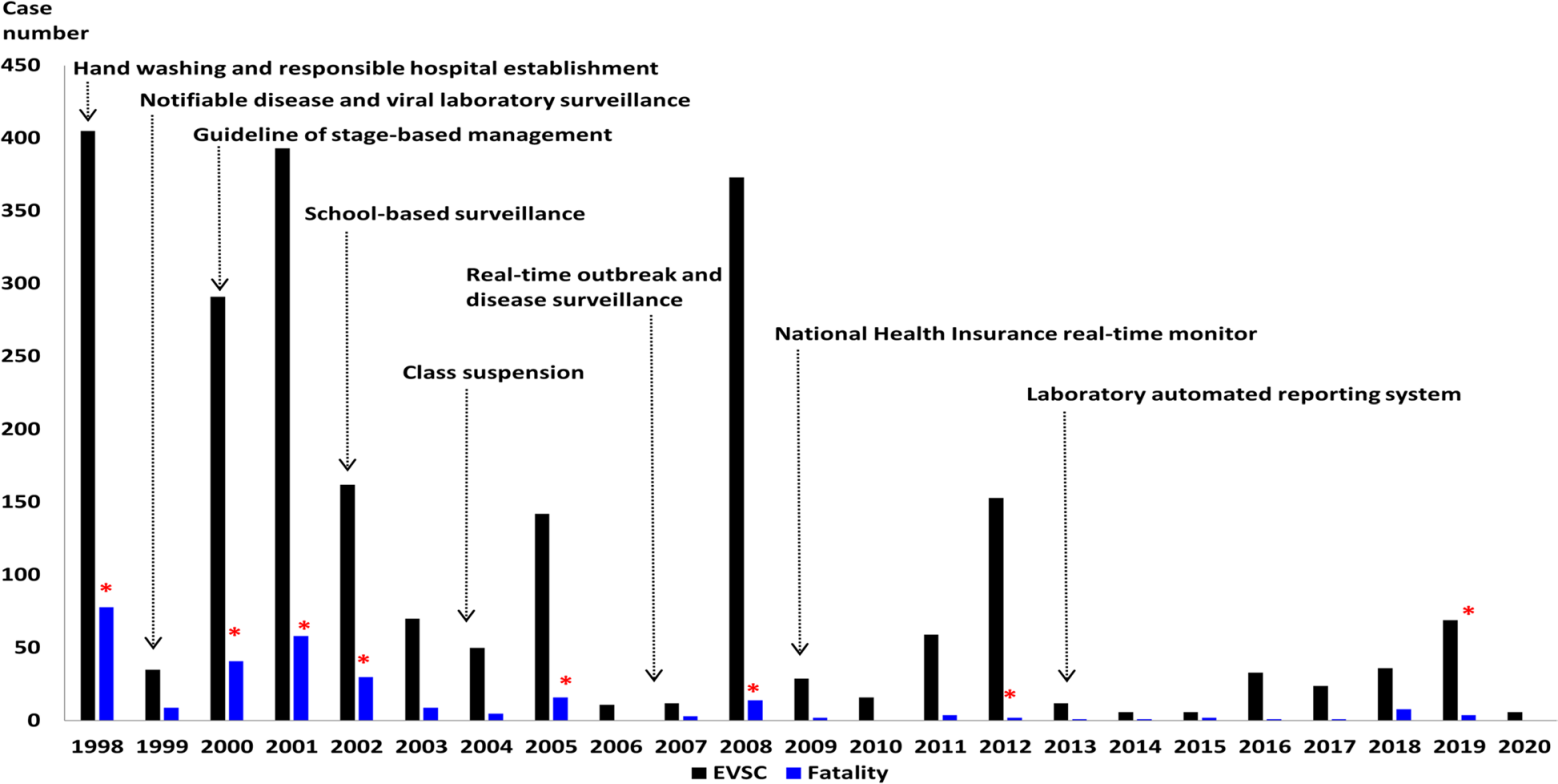
Number of severe or fatal enterovirus infection and the implementation of surveillance and preventive measures, 1998–2020. The numbers of enterovirus infection with severe complications (EVSC) cases and fatality showed a downward trend within the past 20 years. When EV A71 accounted for more than 10% of the annually predominant serotypes (marked with *), both the annual number of EVSC cases and the annual number of fatalities were significantly higher than those in the years when EV A71 accounted for less than 10%

The distributions of case numbers of EVSC and fatalities along with the disease surveillance systems and infection control policies implemented between 1998 and 2020 in Taiwan are summarized in Fig. 4. Most non-pharmaceutical interventions, including disease surveillance systems, hand washing, case isolation, class suspension, and establishment of a medical network for EVSC, had been implemented after 2012. The mean incidences of EVSC were 7.96 and 1.49 per 100,000 children under 6 years of age between 1998 and 2012 and after 2013, respectively (*p* < 0.001). The mean mortality rates were 1.03 and 0.16 per 100,000 children under 6 years of age between 1998 and 2012 and after 2013, respectively (*p* < 0.001). The laboratory automated reporting system was established in 2013, and the case numbers, incidence of EVSC, and fatalities were further reduced thereafter (*P* < 0.001 for all).

### Enterovirus serotype distribution and correlation of different serotypes with severe cases in different years

**Fig. 5:**
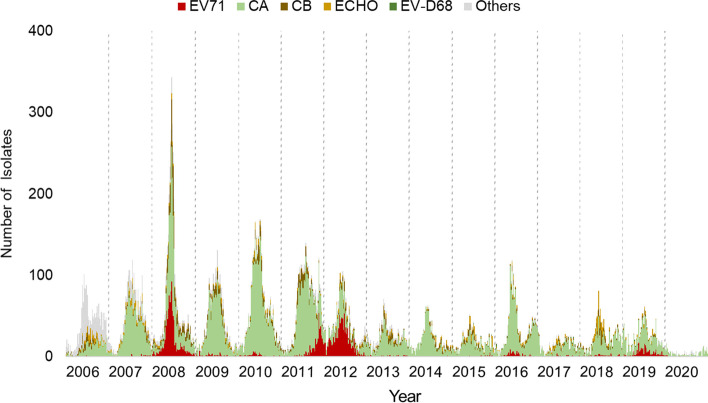
Enterovirus virus isolates according to contracted virology laboratories, 2006–2020. The Taiwan CDC integrated viral contract laboratories for enterovirus isolation since March 1999, and data integration was completed in 2006. The number of EV A71 isolates decreased progressively, and few A71 enteroviruses were isolated after 2012. The average annual positive rate of enterovirus detection was 12%. The mean percentage of EV A71 was 9.1% annually

The weekly numbers of all the enterovirus isolates and EV A71 cases from the viral contract laboratories are shown in Fig. 5. The annual positive rate of enterovirus detection in the virological laboratory surveillance systems was 12% on average. The number of EV A71 isolates decreased progressively, and fewer EV A71 cases were isolated after 2012. The total number of enterovirus isolates was 1732 per year on average, but there were only 221 EV isolates and five EV A71 isolates in 2020 when the COVID-19 pandemic occurred.

The predominant serotypes in the community and in patients with severe complications and fatalities and the severe case-fatality rate in different years are listed in Table 1. The severe case-fatality rate ranged from 0 to 33.3% and declined over time, although there was no significant difference (*p* = 0.065). EV A71 accounted for the majority of EVSC and fatal cases during our study period. The overall proportion of EV A71 among all EV isolates was 11.3% between 2005 and 2020. EV A71 accounted for more than 10% of the annual total EV isolates in 1998 (37%), 2000 (35%), 2001 (31%), 2002 (15%), 2005 (18%), 2008 (27%), 2012 (39%), and 2019 (17%). The number of severe and fatal cases and the incidence of EVSC and mortality rate were significantly higher when EV A71 accounted for more than 10% of the annually predominant serotypes (*p* < 0.05) than when EV A71 accounted for less than 10%. When EV A71 accounted for more than 10% of the annually predominant serotypes, the median number of annual EVSC cases was 226.5 (interquartile range 150.4, 378), and the median number of annual fatalities was 23 (interquartile range 34.5, 45.3); otherwise, the median number of annual EVSC cases was 24 (interquartile range 12, 35.5), and the median number of annual fatalities was 2 (interquartile range 1, 4.5) (both *p* < 0.05).

**Table 1:** Predominant serotypes among enterovirus infection patients with severe complications and deaths

Year	Predominant Serotype Circulating in The Community	Case Number of Enterovirus Infection Patients with Severe Complications	Predominant Serotypes in Enterovirus Infection Patients with Severe Complications	Number of Deaths	Predominant Serotype in the Deceased	Severe case fatality rate
1998	NA	405	EV71	78	EV71	19.30%
1999	NA	35	Cox B3	9	Cox B3	25.7%
2000	NA	291	EV71	41	EV71	14.1%
2001	Cox A16 EV71	393	EV71	58	EV71	14.8%
2002	ECHO6	162	EV71	30	EV71	18.5%
2003	Cox A16	70	EV71	9	EV71	12.9%
2004	Cox A4	50	EV71	5	EV71	10%
2005	Cox B3	142	EV71	16	EV71	11.3%
2006	Cox A2	11	EV71	0	–	0%
2007	Cox A6	12	EV71	3	EV71	25%
2008	Cox A2	373	EV71	14	EV71	3.8%
2009	Cox A6	29	EV71	2	EV71	6.9%
2010	Cox A16	16	EV71	0	–	0%
2011	Cox A10	59	EV71	4	EV71	6.8%
2012	EV71	153	EV71	2	EV71	1.3%
2013	Cox A6	12	EV71	1	ECHO30	8.3%
2014	Cox A10	6	Cox A	1	ECHO11	16.7%
2015	Cox A16	6	Cox B5	2	Cox A16 Cox B5	33.3%
2016	Cox A10	33	EV71	1	EV71	3%
2017	Cox A4 Cox A6	24	EVD68	1	CoxB3	4.2%
2018	Cox A10	36	ECHO11	8	ECHO11	22%
2019	Cox A10	69	EV71	4	EV71	5.8%
2020	Cox A6	6	EV71	0	–	0%

## Discussion

In this study, the case numbers of EVSC and fatalities showed a notably downward trend within the past two decades. We also demonstrated that the incidence of EVSC and mortality rate decreased significantly after 2012, when the majority of the preventive measures had been implemented, and less EV A71 circulated after 2012. The EV A71 seroepidemiological investigation in Taiwan disclosed that the seropositive rate of young children under 6 years of age was below 10% in 2017 [22]. Moreover, the seropositive rates of people less than 20 years of age in 2017 were significantly lower than those in 1997, 1999 and 2007 [22]. This information indicates that the incidence of EV A71 infection in Taiwanese children decreased notably and supports our findings.

The case numbers of EVSC and fatalities were higher when EV A71 accounted for more than 10% of the annually predominant serotypes. Disease and viral surveillance systems played an important role in the early detection of enterovirus infections in the community and medical institutions. Therefore, infection control and preventive measures could be implemented in a timely manner to prevent or contain enterovirus outbreaks. In addition, medical network and education programmes improved the quality of medical care for patients infected with enterovirus. EVSC could be detected early and people could seek prompt medical care due to alert awareness of the warning signs. A practical treatment guideline of stage-based management reduced the case fatality rate of EV A71-related cardiopulmonary failure [20], whereas delayed medical intervention has been proved to be associated with severe infection [23]. All of these implementations contributed to early interventions, which decreased the case-fatality rate and improved prognosis.

One retrospective study provided evidence that school closure was associated with a reduction in the HFMD transmission rate in Singapore [24]. We believe that class suspension had a positive effect on the prevention of enterovirus outbreaks. Our previous study showed that the enterovirus attack rate was strikingly high in kindergarten students because preschool children did not precisely follow the rules of hand hygiene and droplet precautions [25].

Hoang et al. found that the incidence of EV A71 infection among household contacts was 47.6% [11]. Another study showed that the overall EV A71 transmission rate due to household contact was 52%, and viral transmission occurred most often among siblings and cousins [26]. However, the majority of adult household members seldom had obvious clinical symptoms even though they could transmit the virus [11, 26]. Furthermore, EV A71 could survive on a contaminated object or environmental surface for 3 days with 90% relative humidity [27]. Therefore, the promoting correct ways of cleaning and disinfecting the environment, such as using chlorine bleach, might also decrease household transmission rates.

The transmission routes of enteroviruses include droplets, contact and faecal-oral. Previous studies showed that frequent hand washing among school-aged children and their care-givers provided a high protection rate against enterovirus infection [7]. Moreover, cleaning water faucets after hand washing was found to be a protective habit that reduced the risk of complications [23]. The importance of hand washing had been promoted since the EV A71 outbreak in 1998. However, hand washing alone cannot prevent enterovirus transmission. It was interesting to find that the spread or transmission of enteroviruses declined because the entire population strictly followed the rules of hand washing, mask wearing and cough etiquette during the severe acute respiratory syndrome epidemic in 2003 and the COVID-19 epidemic in 2020–2021.

In our study, the mean positive rate of enterovirus isolation from the community contract laboratory was 12%. The total number of enterovirus isolates, on average, was 1732 per year, which decreased to only a total of 221 enterovirus isolates with five EV A71 isolates in 2020. Regarding the clinical aspect, outpatient and emergency department visits for enterovirus infection decreased to less than 2500 per week in 2020. Although there are no available antiviral drugs and vaccines in Taiwan, EV A71 has successfully controlled by using non-pharmaceutical interventions. Owing to well-established surveillance systems, public health awareness, and improvements in infection control measures, the disease burden of EV A71 infection decreased in Taiwan year by year. However, EV A71 epidemics still occur in some countries such as China, Vietnam, and Thailand.

One systemic review and meta-analysis of EV A71-related HFMD used the epidemiologic data from Singapore, Vietnam, South Korea and China [28]. This meta-analysis reported a pooled case fatality rate of 1.7%, with the range varying from 0.4 to 7.7% among different regions [28]. EV A71 infection led to a high case fatality rate (more than 60%) during the Cambodia outbreak in 2012 [5]. During the large EV A71 outbreak in Taiwan in 1998, the estimated case-fatality rate was 44.4 per 100,000 among children younger than 4 years of age, with the highest rate of 96.96 per 100,000 in infants aged six to 11 months [17]. There was a statistically insignificant downward trend of the severe case fatality rate in the following years. Formalin-inactivated EV A71 vaccines were found to be safe and to elicit a strong neutralizing antibody response against EV A71 currently circulating in Asia [29]. It is worth looking forward to further breakthroughs in EV A71 infection control after efficacious vaccine launches in countries in the Asia-Pacific.

There were some limitations to our study. First, we did not analyse the patients’ clinical presentations and risk factors for EVSC and fatality. Second, there were changes in personal hygiene behaviour and environmental sanitation. Improvements in public education may also decrease the risk of enterovirus transmission. However, these confounding factors are difficult to evaluate. Third, there were no fatal cases and few case numbers of EVSC in some years during the study period, and this might have impacted the evaluation of the change in the severe case fatality rate among different years and between enterovirus serotypes.

## Conclusions

Owing to enterovirus A71’s high infectivity and threat to children’s health, Taiwan established multiple surveillance systems and a medical network for enterovirus infection with severe complications, promoted public health education and implemented preventive measures. These measures led to a significant decrease in the numbers of severe and fatal cases of enterovirus in Taiwan from 1998 to 2020. We would like to share the experiences that all of these preventive measures benefitted the control of EV A71 outbreaks and improved patient clinical outcomes. Although the EV A71 vaccine is expected to provide good efficacy and to be another milestone for epidemic eradication, continuous surveillance and the strengthened implementation of infection control policies will still be needed in the future.

## Data Availability

The data that support the findings of this study are available from the Taiwan Centers for Disease Control. The investigators of the original studies retain ownership of their data. Data are available with permission from the Taiwan Centers for Disease Control with investigator support and, after approval of a proposal, with a signed data access agreement. Please contact the corresponding author, Dr. Luan-Yin Chang, if the data of this study are requested.

## Abbreviations

EV: Enterovirus
EVSC: Enterovirus infections with severe complications
HFMD: Hand, foot and mouth disease
CDC: Centers of Disease Control
RODS: Real-time outbreak and disease surveillance system
AAPC: Average annual percent change
